# Digital society generates new challenges on Child Welfare Services

**DOI:** 10.3325/cmj.2017.58.80

**Published:** 2017-02

**Authors:** Heidi Aarum, Hansen, Siv Britt Björktomta, Anna Lydia Svalastog

**Affiliations:** 1 Department for Health and Social Studies, Řstfold University College, Halden, Norway *hah@hiof.no*; 2 Department of Sociology, Uppsala University, Uppsala, Sweden; 3 Centre for Research Ethics and Bioethics, Uppsala University, Uppsala, Sweden

Digital society has created a new situation that challenges the present discourse on public services. Since it is only a recent phenomenon, digital society has not yet been included in the broader filed of social work education and practice. In the present text, we focus on casework with children. The examples described in the text are taken from Scandinavian experiences and reflect our background and practice in social work with children. However, we dare to say that the situation is more or less the same in the rest of Europe, as illustrated by the presented social work examples and references from wider European context.

Because digital society represents a fundamentally new situation for people and social institutions (1,2), there is a need for a paradigmatic shift in its understanding in the field of social work. It is urgent that we generate new concepts that reflect the new situation and develop new analyses and strategies both in social work education and social work practices. The changes that digital environment generated in society need to be understood in all areas of public service, including the social child welfare services. In this article, we discuss the impact of this shift and ways to provide relevant services adjusted to the new needs generated by digital society. Our aim is to start a discussion on the effects of digital society on social work practice and education, with a particular focus on casework. We argue that the concepts of Knowledge Landscape (3,4) and black holes (5) can be helpful in identifying particular challenges that digital society generates for social work in general and social child welfare work in particular, especially for casework concerning family, children, and youth.

## Children and youth in digital society

Digital society has made digital communication and information flow a vital part of everyday life, although access to the Internet and digital technologies varies around the globe. In western societies, this information flow affects all aspects of society, from private interaction through online social networks to public communication to financial transactions and so on. Patient organizations and non-governmental organizations for young people are no exceptions. They also use digital services to communicate and interact with ‘the public’ and different interest groups. Various children’s organizations and organizations disseminating information to children are already well-established online. This corresponds with what we know about children and their online presence. Several studies have shown that children develop online competence as early as first grade (6,7). In Sweden, ever more children tend to use internet on a daily basis, and the number of small children aged less than 4 years who use Internet increases (8).

Two Norwegian organizations for children and youth that actively use the Internet to disseminate information online are Trygg Oppvekst (9) and Juba (10). Trygg Oppvekst, or ‘Safe Upbringing’, is a program based on Antonovsky’s theory on how to prevent harm, ie, salutogenesis. Juba is a youth organization that works on reduction of the use of alcohol and drugs in society, teaches children about democracy, encourages their social participation, and promotes equality.

Other examples are telephone help-lines, addressing different issues and age groups. In the area of mental health in Norway, there is a webpage psykiskhelse.no (ie, ‘mental health’) listing organizations with telephone help-lines and relevant organization’s websites (11). In the field of social work, e-therapy for adults is becoming increasingly available. E-therapy is defined as mental health services offered by mental health professionals, including family social workers, via the Internet. Similar to the use of telephones, e-therapy is used to supplement face-to-face care (12).

A third variant of the digital social work is conducted within the DIPEx International (13). DIPEx is an organization that develops research-based interviews, including young people experiences related to their health problems, and present them online on national homepages. Most interviews are video-interviews, some are sound-based interviews, and some are anonymized texts. DIPEx is an Oxford initiative and an example of a research-based organization that has developed online sources on somatic and mental health, including sexual health, alcohol, and drugs. For example, DIPEx UK (14) offers so-called Health-talk. In the “Young people” category, it provides about 33 interviews with young people on topics such as drugs and alcohol (15).

## Black holes and knowledge landscapes describing risks in digital society

Digital society has made online social networks part of children’s and young people’s daily life. Discussions in the media and between adults and children focus on when rather than if children should have a mobile phone or a social media account. Online bullying, sexual abuse, child pornography, and identity thefts are among the threats that digital society generates for children and young people (16,17).

Many parents underestimate the risks that children are exposed to online (16). There is a discrepancy between what the parents think their children do online and what children actually do. This may include meeting in real life people they met online or seeing, receiving, or sending sexual messages. Due to the social media and the way they are integrated into the lives of children and young people, understanding digital society needs to be included as relevant knowledge in the field of social work education, practice, and research. The impact of social media alters the present discourse on social work services in public sector.

Online social networks represent immediate contact with other network users and new ways to share experiences, feelings, words, sounds, and images. Children and young people can be more versed in digital communication than adults, know more about the variety of platforms, applications, online trends and so on. Still, their understanding of the impact and consequences of social media may be naďve and incomplete. Bullying, social exclusion, revenge-posting, sexual abuse, and online impersonation are all topics known through media and police reports. These problems are also discussed in different social work educational settings. It is an adult responsibility to know about precautionary strategies and who to contact in problematic situations.

Children and young people’s fluent use of communication technology (sound, visual, text) is an appropriate topic for Knowledge Landscape, concentrated specifically on the use of communication tools among children (3).

Although this might be a limited landscape where they only find information on how to install and use new internet tools, it can be as well a larger landscape containing knowledge needed to approach or respond to different users on many diverse platforms, entering open and ‘hidden’ parts of the web, hiding tracks, and hacking into closed resources. Children’s perspective and understanding of the risks and consequences in digital society differs from other Knowledge Landscapes designed for adults, professionals and non-professionals alike. From this perspective, one could say that children and young people and adults and professionals approach the digital society from different perspectives, each having only partial insights, and thus need one another to better understand digital society, the knowledge landscapes involved, and subsequently, the related risks and how to avoid them.

The concept of black hole can be helpful to identify risks and adult responsibilities (5). Black hole is the online isolated field of communication where critical views and transparency are avoided. It is used to describe relatively closed social networks representing particular viewpoints on health (anti-vaccination), society (Anders Behring Breivik, white supremacy, and similar), religion (apocalyptic visions in relation to on-going incidents, for example, the last Ebola breakout or AIDS in the 1980s). Through specific black-hole geography of the Knowledge Landscapes, they push forward their worldviews despite that what they advocate was never accepted as knowledge as it cannot stand the test of critical analyses.

## New psychosocial environment

Children and young people use new technology to socialize with known and unknown people. Therefore, the online environment of information exchange and social dynamics need to be taken into account in the evaluation of children’s psychosocial health and environment and integrated in social work’s education.

Online communication is not completely new to social workers, but for those who do casework and need to get in contact with users of social services, practice has so far been restricted to emails and text messengers, and has not included social media. Children’s access to social networks could become a resource, a part of new practice in social work facilitating communication with children. Today, a problem in casework with children is that professionals’ definition of children’s use of public services is rather limited and communication is, thus, controlled through contact with adults, ie, parents (18). Both Skandinavian and international research shows that direct dialogue between social workers and children declined over the last decade (18-20).

It is especially problematic in casework when professionals lack direct contact with children, because we know that children’s and parents’ narratives and experiences do not always coincide. The child’s own perception of the situation may not always be heard and included in social records and reports (19). This collides with the European consensus on the importance of inclusion of the child’s perspective (20). To use social media in social work practice could help social workers ensure inclusion of children’s own narratives, while still keeping within the present legal and ethical frameworks.

In social work with adults, the use of e-therapy is an example of how social workers use online communication for purposes other than gathering information or sending emails and text messages. Research on e-therapy shows that many families see it as an advantage to be able to express thoughts and feelings in writing through various Internet contacts (12). This may also be a good way to communicate with young people in general.

Browsing the Internet or asking friends questions via social media differs from going to a library or asking a physician, psychologist, priest, or Elders of Indigenous communities in person. Online search provides different results from the good old-fashioned search of encyclopedias at home or library. Online results draw information from a variety of contexts. An online search produces a plethora of results, some of which are problematic, because search algorithms are based on previously made individual choices. The particular challenges concerning children and young people are Facebook algorithms and applications especially made for children, which have established new ways of communicating and may be used by pedophiles to get in contact with children (21,22).

The complexity of online life – interaction, communication, and search for knowledge – does not automatically mean that what is found or distributed online is incorrect or bad. It does mean, however, that communication and knowledge are different in ways that social workers need to understand and social work education needs to include. Different theories of texts and hermeneutics can help develop this further, such as Paul Ricoeur’s understanding of text, which emphasizes how a text, in addition to being interpreted, also carries a meaning and, as such, also represents resistance and limits regarding how and what the text can be said to represent or contain (23).

## What a social worker needs today

First, social workers need to understand the effects of digital society, in particular Internet and social media, on children and young people and the ways they communicate, interact, and socialize. Second, they need to understand the risks related to the use of social media in order to be able to teach children and young people how to use and how not to use the Internet. Third, they need to be informed by children and young people to create relevant services for them. And last, social workers need to develop new methodologies of working with children and young people to meet the challenges of digital society, ie, to supporting children as actors and protect them against harm.
